# In Silico Approaches to Identify Polyphenol Compounds as α-Glucosidase and α-Amylase Inhibitors against Type-II Diabetes

**DOI:** 10.3390/biom11121877

**Published:** 2021-12-14

**Authors:** Jirawat Riyaphan, Dinh-Chuong Pham, Max K. Leong, Ching-Feng Weng

**Affiliations:** 1 Rubber Authority of Thailand, Bangkok 10700, Thailand; jirawat.riyaphan@gmail.com; 2 Biomaterials and Nanotechnology Research Group, Faculty of Applied Sciences, Ton Duc Thang University, Ho Chi Minh City 700000, Vietnam; phamdinhchuong@tdtu.edu.vn; 3 Department of Chemistry, National Dong Hwa University, Hualien 97401, Taiwan; 4 Functional Physiology Section, Department of Basic Medical Science, Xiamen Medical College, Xiamen 361023, China

**Keywords:** polyphenol, in silico, herb medicine, type II diabetes

## Abstract

Type-II diabetes mellitus (T2DM) results from a combination of genetic and lifestyle factors, and the prevalence of T2DM is increasing worldwide. Clinically, both α-glucosidase and α-amylase enzymes inhibitors can suppress peaks of postprandial glucose with surplus adverse effects, leading to efforts devoted to urgently seeking new anti-diabetes drugs from natural sources for delayed starch digestion. This review attempts to explore 10 families e.g., *Bignoniaceae*, *Ericaceae*, *Dryopteridaceae*, *Campanulaceae*, *Geraniaceae, Euphorbiaceae*, *Rubiaceae*, *Acanthaceae*, *Rutaceae,* and *Moraceae* as medicinal plants, and folk and herb medicines for lowering blood glucose level, or alternative anti-diabetic natural products. Many natural products have been studied in silico, in vitro, and in vivo assays to restrain hyperglycemia. In addition, natural products, and particularly polyphenols, possess diverse structures for exploring them as inhibitors of α-glucosidase and α-amylase. Interestingly, an in silico discovery approach using natural compounds via virtual screening could directly target α-glucosidase and α-amylase enzymes through *Monte Carto* molecular modeling. *Autodock*, *MOE-Dock*, *Biovia Discovery* Studio, *PyMOL,* and *Accelrys* have been used to discover new candidates as inhibitors or activators. While docking score, binding energy (Kcal/mol), the number of hydrogen bonds, or interactions with critical amino acid residues have been taken into concerning the reliability of software for validation of enzymatic analysis, in vitro cell assay and in vivo animal tests are required to obtain leads, hits, and candidates in drug discovery and development.

## Highlights

In silico approaches can rapidly provide evolving experimental and analytical tools to identify polyphenol plant families for treating T2DM.

In silico studies can determine polyphenols as putative inhibitors of α-glucosidase and α-amylase enzymes regulating blood glucose in T2DM.

In silico modeling accelerates screening of a huge database in a high throughput fashion to facilitate drug discovery and development.

## 1. The Impact of T2DM

Diabetes mellitus (DM) is a medical condition characterized by metabolic and chronic disorders with abnormal levels of carbohydrate, protein, lipid, and electrolysis metabolism, resulting in loss of control over blood glucose level [1,2]. Clinically, DM can be categorized into four subtypes: type 1 DM (T1DM), which was formerly known as insulin-dependent DM (IDDM) or juvenile-onset DM and is primarily resulted from pancreatic β-cell destruction and diagonized by absolute insulin deficiency; type 2 DM (T2DM), which was formerly known as noninsulin dependent DM (NIDDM) or adult-onset DM and is predominantly characterized by insulin resistance with relative insulin deficiency or secretory defect with insulin resistance; gestational DM (GDM), in which women are diagnosed as diabetic during pregnancy, and other specific types of diabetes that were not included in any previous forms according to the American Diabetes Association (ADA) [3].

The prevalence of diabetes in the world was estimated to be 2.8% for all ages in 2000, and that is expected to increase to approximate 4.4% in 2030 [4]. Diabetes causes of death will increase to 366 million by 2030 [5]. The World Health Organization (WHO) estimates that 415 million people will be affected by diabetes in 2015 [6], and this is expected to rise to 642 million by 2040, worldwide [7]. Currently, the numbers of diabetic patients has significantly increased in the population between 45 and 64 years of age in many countries, particularly in China, India, and Southeast Asia [8,9,10]. Of various DM subtypes, T2DM, which is characterized by chronic metabolic imbalance [4], beta-cell failure and insulin resistance, and can be alleviated by changing lifestyle by dietary control and exercise [11], is the most common type, accounting for more than 90% of all DM patients. The onset of T2DM can be attributed to behavioral, environmental, and genetic factors, leading to insulin resistance and deficiency [11,12,13,14]. Importantly, the involvements of several factors in T2DM that cause resistance of target tissues to insulin, usually resulting from abnormal insulin secretion [15]. T2DM is a common and increasingly prevalent disease and is a major public health problem worldwide [16].

The clinical diagnosis of T2DM is reliant on one of four plasma glucose (PG) evaluations: (i) fasting plasma glucose (FPG) (>126 mg/dL); (ii) 2-h 75-g oral glucose tolerance test (OGTT) (>200 mg/dL) [4]; (iii) random PG (>200 mg/dL) with symptoms of hyperglycemia, or (iv) hemoglobin A1C level >6.5% [17]. Furthermore, human subjects are considered as prediabetics when their FPG is above the normal value but less than the threshold, namely 110–126 mg/dL, and they are predispose to diabetes, insulin resistance, and a higher risk of cardiovascular (CV) and neurological pathologies [18,19].

## 2. T2DM Medicines

A healthy lifestyle and drug treatments are common practices in controlling blood glucose levels and delaying or preventing the occurrence of complications in T2DM patients [20]. Insulin treatment is available in clinics as are innovative T2DM therapy agents that may be applicable for patients based on various molecular targets and pathways. Specific inhibitors include α-glucosidase, sodium glucose linked transporter-2 (SGLT-2), dipeptidyl peptidase 4 (DPP-4), peroxisome proliferator activated receptor-γ (PPARγ), insulin receptor kinase (IRK), and glucose transporter 4 (GLUT4). A G protein-coupled receptors (GPCR) such as the GLP-1 receptor inhibitor blocks G protein (heterotrimeric) production (Table 1) [17]. To date, all available T2DM medicines are associated with various side effects such as digestion disorder, increased risk of heart failure, infection of the urinary tract, nerves, kidneys, and eye damage [9]. For instance, metformin, which is the most prescribed oral therapeutic agent to treat T2DM in western counties and Japan [21], can cause gastrointestinal side effects, such as loss of appetite, diarrhea, nausea, vomiting, flatulence, and abdominal pain [22].

Folk or herbal medicines as traditional medicines or traditional Chinese medicines (TCM) have been used as botanical products or compounds for many years, and some of them have been derived from crude extraction [23]. Some have shown the potential as therapeutic agents against T2DM and other disease conditions [18]. The necessity for developing therapeutic drugs with fewer side effects is still unmet, due to limited efficacy or unacceptable disadvantages including side effect sand drug resistance in current available therapeutic agents [24]. Antidiabetic properties of more than 1200 plants have been asserted and, using these, the adverse effects and inflammation associated with the most common drugs can be reduced [25].

## 3. Polyphenols & Plant Families

Polyphenols, which are natural compounds and can be extracted from common plants, have been a subject of considerable research interest in recent years because of their implications in the treatment of various diseases such as DM and human health-related disorders [23].

Several plant families have been investigated for their anti-hyperglycemic abilities [47]. Recently, polyphenol-rich functional foods have been proposed as supplementary and nutraceutical treatments for T2DM [48]. It has been demonstrated that polyphenolic compounds, which contain multiple phenolic moieties such as lignans, stilbenes, flavonoids, phenolic acid, hydroxycinnamic acids, hydrobenzoic acids, and olive oil polyphenolics [19], can result in antioxidation and anti-inflammation, and mediate enzymatic metabolism to moderate and decrease glucose absorption in the intestine [49]. For various reasons in recent years, traditional plant and herb therapies prescribed in the indigenous system of medicine [50], with different mechanisms [51], have commonly been used. 

### 3.1. Euphorbia thymifolia Linn. (E. thymifolia)

*Euphorbia thymifolia* Linn. (*Euphorbiaceae*), commonly known as *laghududhika* or *choti-dudhi*, is a prostate annual herb [52]. Their leaves, seeds and fresh juice of the whole plant are used as a stimulant and astringent in worm infection [53]. It has been reported that plant extracts can be used as traditional medicines to treat various disorders in many parts of the world [54], using plants such as cassava (*Manihot esculenta*), castor oil plant (*Ricinus communis*), Barbados nut (*Jatropha curcas*), and the Para rubber tree (*Hevea brasiliensis*) despite the fact that many of them are grown as ornamental plants such as poinsettia (*E. pulcherrima*), leafy spurge (*E. esula*), and Chinese tallow (*Triadica sebifera*). Euphorbiaceae species have been used by different populations as folk medicines for remedying a broad range of diseases and complaints, including cancer, diabetes, diarrhea, heart diseases, hemorrhages, hepatitis, jaundice, malaria, ophthalmic diseases, rheumatism, and scabies [55], with some disadvantages including drug resistance to the plants’ components [56]. The hypoglycemic potential of this plant family was mainly identified by virtual screening for high binding energies (4.8–9.9 Kcal/mol) and strong hydrogen interactions. Moreover, insulin levels were significantly increased and the lipid profile and body weight were improved after 20 days when an ethanolic extract of *R. communis* (Euphorbiaceae) at 500 mg/kg p.o. was administrated to those diabetic rats [57]. Recently, some herbal medicines have been reportedly to treat T2DM in worldwide studies, and some of their functions as α-glucosidase and α-amylase inhibitors to exert their anti-hyperglycemia efficacy have been identified [58], since inhibition of intestinal α-glucosidases can limit postprandial glucose levels by delaying the process of carbohydrate hydrolysis and absorption, making such inhibitors useful for the management of T2DM. Plants and microorganisms are rich sources of α-glucosidase inhibitors. For example, acarbose, 1-deoxynojirimycin, and genistein were originally isolated from natural sources [59]. *E. hirta* L., which is a traditional plant used for various disease treatments, has been under investigation as an α-glucosidase inhibitor. *Triphala*, which is a combination of *Terminalia chebula*, *T. belerica*, *E. officinalis*, is under in vivo evaluation for antidiabetic potential in relation to antioxidant activity [60]. *T. belerica* was found to be most active in reducing serum glucose levels followed by *E. officinalis*, *T. chebula*, and *Triphala*, which is a combination of all the three products, significantly reducing hyperglycemic effect in alloxan-induced diabetic rats [61]. Aqueous extracts of *E. hirta* L. showed inhibition of α-amylase activity compared to acarbose [62]. In contrast, α-amylase inhibitors from plant sources have a lower effect against α-amylase activity and stronger inhibition of α-glucosidase activity [63].

### 3.2. Bignoniaceae

*Bignoniaceae* are woody, trees, shrubs, and lianas found in all tropical floras of the world, with lesser representation in temperate regions, and belong to a family of flowering plants in the order *Lamiaes*, commonly known as the bignonias [64]. *Bignonieae* comprise a major component of neotropical liana flora. Most other species are woody shrubs and trees including savannah and tropical forest canopy trees, although these three groups have adopted a herbaceous habit, mostly at high elevations in the Himalaya (*Incarvillea*) and the Andes (*Arggylia, Tourrettia*) [65,66]. Interestingly, *Tecona stans* (L.) Juss ex Kunth plants are extensively used for empirical DM treatment, but their antidiabetic mechanisms remain to be clarified [67]. This family of compounds may show their antidiabetic effect by stimulating glucose uptake in T2DM [68].

### 3.3. Ericaceae

The *Ericaceae* are dominant plants of acid heathlands and upland soils, and include the genera *Calluna, Erica, Vaccinium, Azelea, Rhododendron,* and the *Epacrids* of Australasia, which grow in dry sandy soils [66]. The *Ericaceae* are a family of flowering plants, commonly known as the heath or heather family, and can be found most commonly in acid and infertile growing conditions [69]. Wan and Shou (2013) observed that a crude extract from *Vaccinium corymbosum* (*Ericaceae*), including a phenolic compound, shows powerful α-glucosidase inhibitory activity and it is even more efficacious than the marketed drug acarbose [70]. Moreover, the α- and β-glucosidases inhibitory activities of *Rhododendron arboreum* (*Ericaceae*) have been investigated by various in vitro studies and the results suggest that it is a potent α-glucosidase inhibitor with an IC_50_ of 3.3 ± 0.1 μM, many-fold higher than that of acarbose [71]. A wide variety of phenolic compounds, which are the most abundant secondary metabolites of plants with more 8000 phenolic structures, have great potential in protecting against cardiovascular diseases, diabetes, cancer, and obesity [72].

### 3.4. Dryopteridaceae

Many of the *Dryopteridaceae*, which are a family of leptosporangiate ferns in the order Polypodiales, are cultivated as ornamental plants. The fern genus *Dryopter* (*Dryopteridaceae*) is among the most common, and includes 225–300 species worldwide in temperate forests in the northern hemisphere [73]. *Dryopteris cycadina* is a medicinal plant from the *Dryopteridaceae* family. It has been traditionally used as a folk medicine to treat rheumatism, epilepsy, and pain and to remedy snake bites and fungal infections [74]. In vitro studies show that compounds in *D. cycadina* inhibit α-glucosidase in a concentration-dependent manner, further validated by in silico studies, and show strong hydrogen bonds interaction. Four strong interactions between amino acid side chains and hydrogen bonds (Asp215, Asp352, Arg422, and Gln182) are reported [75].

### 3.5. Campanulaceae

*Codonopsis*, belonging to the family *Campanulaceae*, is a genus including 42 species of dicotyledonous herbaceous perennial plants predominantly found in central, east and south Asia. Several *Codonopsis* species are widely used in traditional medicine and are considered to have multiple medicinal properties. It has been shown in phytochemical studies that *Codonopsis* species, which contain mainly polyacetylenes, phenylpropanoids, alkaloids, triterpenoids, and polysaccharides, contribute to multiple biological functions [76]. The less popular *Codonopsis* species remain to be studied and exploited. One of genus, *Lobilia chinensis* has been extracted to obtain two new pyrrolidine alkaloids, radicamines A and B, that are α-glucosidase inhibitors [77]. In addition, *Codonopsis lanceolate* Trautvein is a plant of Campanulaceae family, which is distributed throughout China, Japan, and Korea. The roots of *C. lanceolate* have been cultivated and used as a food. Other Codonopsis species such as *C. pilosula* and *C. tangshen* have been used as medicines (Tang-Sam) for ulcers treatment, memory improvement, and immune stimulation [78,79].Various reports have indicated that the isolated secondary metabolites of *C. lanceolata* roots e.g., triterpenoid, saponin, and alkaloids, show tangshenoside I and β-adenosine with an IC_50_ of 1.4 and 9.3 mM for α-glucosidase inhibition, respectively [80]. It has been demonstrated previously that *Lobelia sessilifolia* can potently inhibit rice α-glucosidase, and crude extracts and coffee beans can be very specific and potent α-galactosidase inhibitors [81]. Zafar and Khan (2016) recently reported that the alkaloids isolated from some of *Campanulaceae* and *Lobelia* species, along with standard acarbose, exert significant anti-glucosidase effects. Strong hydrogen bond binding modes of these inhibitors display four interactions between amino acid side chain and hydrogen bonds (Lys155, Glu304, Arg312, and Asn153) [82].

### 3.6. Geraniaceae

The genus, *Geranium*, which belongs to the Geraniaceae family, is represented by 350 species in the world, of which 38 species include 14 endemic taxons in Turkey [83]. The genus *Geranium* is known to contain flavonoids, tannins, anthocyanidins, lignans, sterols, and polyphenolic compounds, as well as essential oils [84]. *Geraniaceae* are herbs or subshrubs, and a family of flowering plants in the order Geraniales. The extracts of *Geranium graveolens* L. of Geraniaceae are essential oils and act as both α-amylase and α-glucosidase inhibitors [77]. *G*. *wallichianum* has shown very good α-glucosidase inhibition activity. These observations suggest that the presence of potent compounds can inhibit these carbohydrate digesting enzymes. A methanolic extract prepared from the aerial parts of *G. wallichianum* is the most potent agent for inhibition of α-glucosidase, α-amylase, and pancreatic lipase with inhibitions of 65.81%, 72.89%, and 52.80%, respectively. Thus, *G. wallichianum* is a plausible subject for further studies for the treatment and management of metabolic syndrome [85]. Some *Geranium* species have been used to treat diabetes. *G. asphodeloides*, for instance, showed high α-glucosidase inhibitory effect compared with acarbose with an IC_50_ value of 0.85 µM in vitro study [86].

### 3.7. Rubiaceae

The *Rubiaceae* are flowering plants, commonly known as the coffer, or bedstraw family. They consist of terrestrial trees, shrubs, lianas, or herbs that are recognizable by simple, opposite leaves with interpetiolar stipules. Alkaloids, phytosterols, carbohydrate, and saponins extracted from many species of the Rubiaceae family, such as *Gardenia taitensis,* can reduce blood glucose, total cholesterol, LDL and VLDL cholesterol, and improve HDL cholesterol associated with T2DM treatment [77]. Ethanol extracts of leaves and twig of some plants in the Rubiaceae can have 80% inhibitory activity of α-glucosidase [87]. For example, *Xeromphis uliginosa* Retz. is found in root extracts and reduces the blood glucose [88]. The extract from *Morinda tinctoria* fruits shows inhibition of glucose diffusion [89]. The leaves and root extracts of *Nauclea latfolia* Sm lower fasting blood glucose, increase MCV and MCH, reduce iWBC and increased lymphocyte levels. A stem bark extract of *Neolamarckia cadamba* shows antihyperglycemic activity [90]. An extract of *Anthocephalus indicus* leaf can reduce blood glucose and total cholesterol, triglycerides, HDL and LDL [91]. These families may be used as diabetic herbal treatments. *Rubia cordfolia* Linn. from root extracts acts as an α-amylase and α-glucosidase inhibitor [92]. Furthermore, oral administration of *Hamelia patens* (*Rubiaceae*) exhibited the greatest inhibition of α-glucosidase in an in vivo test. *H. patents* inhibits α-glucosidase activity as a traditional medicine dur to its active compounds [93]. Methanol extracts of *Hedyotis biflora* L. (*Rubiaceae*) showed 50% inhibition of α-glucosidase at a concentration of 480.20 ± 2.37 µg/mL in in vitro tests [94]. *Nauclllea latifolia* also belongs to *Rubiaceae* family and the root stem is traditionally and empirically used by diabetic patients in Benin to manage glycemia [95]. In in vivo studies, *N. latifolia* (Rubiaceae) was assessed for lowering fasting blood glucose in normoglycaemic and streptozotocin (STZ)-diabetic rats at the highest administered dose (400 mg/kg) and lowered the fasting blood glucose of the diabetic rats by 31.7% (aqueous extract) and 36.1% (ethanolic extract), respectively. Consequently, this plant can be can have traditional use for treatment of T2DM [96].

### 3.8. Acanthaceae

*Acanthaceae* is a family of dicotyledonous flowering plants. Flavonoids, alkaloids, terpenoids, tannins, and steroids extracted of *Acanthus illicfolius* reduce blood glucose level and result in better regeneration of β-cells [77]. Additionally, extracts from *Justicia secunda* Vahl. leaves used to treat DM symptoms showed inhibitory effects on α-glucosidase, and the potential of *J. secunda* for traditional medicinal use in T2DM treatment was supported [97]. *Justicia* is the largest genus of the Acanthaceae family and consists of approximately 600 species distributed in pantropical and tropical regions. In traditional medicine, the extracts of leaves are used to treat diabetes and diabetic symptoms [98]. Diterpenoid lactones and andrographoloids, including gibenclamide, glimepiride, glipizide, nateglinide, rosiglitazone, pioglitazone, and repaglinide from *Andrographis paniculata* Nees are found to inhibit CYP2C9, CYP2C19, CYP2D6, CYP3A4, and glucose transporter (GLUT4) [99], as well as increasing glucose metabolism and reducing lipid accumulation in differentiated adipocytes [100,101,102]. Moreover, *A. paniiculata.* (Burm.f.) Nees (*Acanthaceae*) when applied in oral carbohydrate tolerance tests with starch (3 g/kg), sucrose (4 g/kg), or glucose (2 g/kg), separately in 18-h fasted rats, resulted in reduced sucrose and starch, similar to an acarbose effect, while it had no peak blood glucose with a suppressive effect after an exogenous glucose load in both normal and STZ-induced diabetes rats [103]. Interestingly, *Clinacanthus nutans* belongs to the Acanthaceae family and is used to treat diabetes in Malaysia. In vitro, this plant was identified as a potential α-glucosidase inhibitor with an IC_50_ lower than 50 µg/mL. In silico, it showed strong hydrogen bonding and some hydrophobic interaction between inhibitors and proteins including Asn259, Hid295, Lys156, Arg335, and Gly209. Additionally, hydrogen bonding is involved in Trp15, Tyr158, Val232, Hie280, Ala292, Pro312, Leu313, Val313, Phe314, Arg315, Try316, Val319, and Trp343 amino acid residues [104]. Previously, this plant was also identified as having potential α-glucosidase inhibition properties. Interaction between inhibitors and protein were predicted involving residues Lys156, Thr310, Pro312, Leu313, Glu411 and Asn415 with hydrogen bonds at Phe314, and at Arg315 with hydrophobic bonding. Hence, α-glucosidase inhibitor has been identified in *C. nutans* leaves, indicating the plant’s therapeutic effect to relieve T2DM [105].

### 3.9. Rutaceae

*Rutaceae*, commonly known as citrus family, is a family of flowering plants with approximatively 160 genera, also having flowering species. The most economically important genera in the family are Citrus, including the orange (*Citrus sinensis*), lemon (*C. limon*), grapefruit (*C. paradisi*), and lime (mostly *C. aurantifolia*) as well as *Zanthoxylum* or *Fagara* and *Agathosma*. Species of the *Fagara* genus have been found to have antimicrobial activities. *Fagara leprieurii* (Guill and Perr) Engl. is used traditionally in cases of gastritis, diarrhea, cancer, ulcer, and kidney ache, as well as other infectious diseases [106]. In this term, finger citron (*C. medica* L. var.) fruits, widely cultivated in Japan; possess insulin secretagogues and slimming effects that would be very beneficial to T2DM patients [107]. The extract of *Clauserna anisate* Bum. f. root was show to stimulate secretion of insulin [108]. Moreover, leaf extracts of *Murraya koeingii* (L.) Spreng can increase glycogenesis, decrease glycogenolysis and gluconeogenesis [109]. After oral administration of pulp extract of *Syzygium cumini* fruit to normoglycemic and STZ-induced diabetic rats they showed hypoglycaemic activity in 30 min, possibly mediated by insulin secretion and inhibited insulin activity of the pancreas [52]. Interestingly, a flavonoid from *Rutaceae aurantiae* inhibits advanced glycation end-products (AGEs) and reduces albumin, that are significantly diminished in flavonoid-treated diabetic rats [110]. In an in vitro study, terpenoids isolated from stem and bark of *Fagara tessmannii* (Rutaceae) showed strong inhibitory activity with an IC_50_ of 7.6 µmol/L, which resembled the inhibitory activity of acarbose which was used as a positive control [111]. Many α-glucosidase inhibitors such as alkaloids, terpenoids, anthocyanin, and phenolic compounds were found to have α-glucosidase inhibitory potency.

### 3.10. Moracea

*Moraceae*, often called the mulberry family of flowering plants, comprises about 40 genera and over 1000 species. The includes *Artocarpus heterophyllus* (Jackfruit), *A. altilis* (Bread fruit), *A. camans* (Bread nut) and *A. integer* (cempedak) which possess antibacterial, anti-inflammation, antioxidant, and antidiabetes properties [112]. Moreover, *Dieffenbachia* *picta* is an herbaceous plant used in southern Cameroon as an antidiabetic and antihypertensive drug [113]. Leaves of white mulberry (*Morus alba*, Moraceae) have been used in traditional medicine to treat diabetes. Recently, leaves and stems were found to inhibit both α-amylase and α-glucosidase activities by at least 50% [114]. Bark extract of *Ficus bengalensis* decreased blood glucose level, restores the levels of serum electrolytes, glycolytic enzymes, and hepatic cytochrome P-450 dependent enzyme systems and decreases the formation of liver and kidney lipid peroxides [115]. Other *F. religiosa* Linn. can cause rising serum insulin and initiate insulin release [116]. Leave from *Morus alba* increased the β-cell number in diabetic islets reduced levels of glycosylated hemoglobin [117], especially decreasing triglycerides and VLDL, and restored elevated levels of blood urea. Besides, this species can protect pancreatic β-cells from degeneration and diminish lipid peroxidation [118]. *M. indica* L. leaf extracts increased glucose uptake [119]. *M. bomoysis* regenerated β-cells of the islets of Langerhans [77]. The oral administration of the extract of *F. bengalensis* caused enhanced serum insulin levels in normoglycaemic and diabetic rats. The increased insulin secretion was mainly due to inhibition of pancreatic insulin activity from the liver and kidney [120]. The blood glucose lowering activity of a dimethoxy derivative of leucocyandin 3-O-beta-d-galactosyl cellobioside isolated from the bark of *F. bengalensis* at a dosage of 250 mg/kg, p.o. in normal and moderately diabetic rats was mainly due to insulinomimetic activity [121]. A glycoside of leucopelargonidin isolated from the bark of *F. bengalensis* demonstrated significant hypoglycaemic, hypolipidemic and serum insulin-raising effects in moderately diabetic rats. Dimethoxy ether of leucopelargonidin-3-O-alpha-L rhamnoside at a dose of 100 mg/kg, p.o. had significant hypoglycaemic and insulinomimetic activity in healthy and alloxan-induced diabetic dogs during a 2 h test [121].

## 4. Potential Polyphenols of 10 Plant Families with Regulation of α-Glucosidase and α-Amylase Activity

α-Glucosidase is located in the brush border of the small intestine and breaks down starch and disaccharides. α-Amylase breaks internal α-1, 4-glycosidic linkages of starch into glucose and maltose in the digestive organs [6]. Amylase is found in saliva glands whereas pancreatic amylase is secreted by the pancreas into the small intestine [7]. However, blood glucose level can be determined by α-amylase via increasing digestion of starch and disaccharides [8]. The therapeutic approach to treating T2DM is to delay absorption of glucose through inhibition of enzymes including α-glucosidase and α-amylase in the digestive organs [15,122]. The mechanisms and therapeutic potential of polyphenols can be used for clinical trials and drug discovery in the management of T2DM. Polyphenols are found mainly in plant-based foods e.g., fruits, vegetables, whole grains, coffee, tea, and nuts. Polyphenols may affect glycemia and T2DM through different mechanisms, such as promoting the uptake of glucose in tissues (α-glucosidase and α-amylase) and improving insulin sensitivity [123]. Besides, polyphenol compounds such as caffeic acid, curcumin, cyanidin, daidzein, epicatechin, eridyctiol, ferulic acid, hesperetin, narenginin, pinoresinol, quercetin, resveratrol, and syringic acid can significantly inhibit the α-glucosidase enzyme. Especially, catechin, hesperetin, kaempferol, silibinin, and pelargonidin are found to be potent α-amylase inhibitors [49]. The current study aimed to investigate polyphenol families to discover a new class of α-amylase and α-glucosidase inhibitors to target these enzymes. Different treatments such as diets and drugs are recommended for α-glucosidase and α-amylase inhibition. Especially, the primary structure of polyphenols can affect the inhibition levels of α-glucosidase and α-amylase enzymes [124]. Various families of polyphenols have beneficial effects and have been shown to suppress α-glucosidase and α-amylase at a 50% inhibition level and higher [125]. The abundant polyphenols flavan-3-ol monomers (catechins), were evaluated against the pharmacological glucosidase inhibitor-acarbose, and catechin 3-galltes strongly inhibited both α-glucosidase and α-amylase activity [126]. Moreover, positive relationships among α-glucosidase inhibitory and the polyphenol content of these 28 edible plants were found in both aqueous and methanolic extracts as well as the fresh juice of the whole plant [127]. Interestingly, some plants show inhibition of both α-glucosidase and α-amylase against T2DM (Table 2).

DPP-4 inhibitors improve β- and α-cell function and decrease glucagon concentrations [208]. Normally, DPP-4 is widely expressed in numerous tissues including endothelial cells of multiple vascular beds, rendering the enzyme highly accessible to peptide substrates circulating through the gut, liver, lung, and kidney [209]. In an in silico study, the DPP-4 active site interacted widely with a hydrophobic pocket via hydrophobic inhibitor moieties such as Try629 and Try547 [210] and also interacted with other proteins and proline (P) and alanine (A) residues [211]. GLP-1, which is a physiological incretin hormone from the lower gastrointestinal (GI) tract [212], is produced from the proglucagon gene in the L cell of the small intestine and secreted in response to nutrients. GLP-1 exerts its major effects by stimulating glucose-dependent insulin release from the pancreatic islets. GLP-1 has also been shown to slow gastric emptying [213] with substantial postprandial GLP-1 release which, in these conditions, interferes with GLP-1 receptor signaling and has a significant impact on glucose regulation after eating, including DPP-4 inhibition [214]. The GLP-1 receptor is a member of family B with G protein-coupled receptors and is an important drug target for T2DM. This hormone docks with high affinity and is a full agonist with specific amino acid residues namely, Arg131, Lys136, Glu133, and Glu125 within the same region of the receptor amino termini [215,216]. GLP-1 plays an important physiological role in maintaining blood glucose homeostasis, and may be a very effective therapeutic drug for the treatment of T2DM [216,217]. Thus, the basis of molecular docking of ligand binding and subsequent activation is clinically important for the GLP-1 receptor [215].

Insulin receptor kinase (IRK) is a heterotetrameric receptor composed of two extracellular α-subunits and two transmembrane β-subunits. Insulin is a hormone responsible for glucose and lipid metabolism. Binding of this hormone to the extracellular domain of the insulin receptor (IR) induces a conformational change that facilitates ATP binding and leads to increased autophosphorylation of the receptor [218]. Moreover, IRK is subsequently autophosphorylated and activated to tyrosine-phosphorylated key cellular substrates that are essential for interacting with the insulin response [219]. IRK activation occurs at the beginning of insulin signaling in the cell surface, while in silico studies have shown insulin-sensitive auto-phosphorylation of receptors with mutated glycosylation sites lacking glycan chains at Asn624-730, Asn730-743 and Asn881, but with a constitutively active tyrosine kinase [220]. At Asn1234, IRK lacking glycosylation exhibited a threefold increase of basal autophosphorylation and played a critical role in signal transduction for IRK activation [221].

Insulin receptor substrate (IRS) molecules are key mediators in insulin signaling. Several polymorphisms in the IRS gene have been identified; however, only the Gly to Arg 972 substitution of IRS-1 seems to have a pathogenic role in the management of T2DM [222]. In the IRS-1 gene, Gly972 with an Arg substitution has shown to be related to insulin resistance in T2DM [223]. Therefore, IRS is an important ligand in the insulin response of human cells; especially, IRS-1 and IRS-2 are ubiquitously expressed and are the primary mediators of insulin-dependent mitogenesis and regulation of glucose metabolism in most cell types [224]. IRS-1 was originally identified as the major substrate of insulin receptor and IGF-1 receptor tyrosine kinase and represents the prototype of the IRS family proteins [225]. IRS-2 contains an additional domain, the KRLB domain, that interacts with the tyrosine kinase domain of the IR and may function to limit IRS-2 tyrosine phosphorylation [226]. In addition, both IRS-1 and IRS-2 have been associated with regulating GLUT4-dependent glucose uptake in the response to insulin [227]. To explore targeting of specific molecules or genes, and further accelerate the drug discovery and development, computational biology associated with virtual screens plays a key role. 

## 5. In Silico Approaches

In silico technologies play an increasingly imperative role in drug discovery and development mainly due to their fast throughput, economical efficiency, and labor saving characteristics [228], especially compared with their in vitro and in vivo counterparts that for identifying new natural compounds as drug targets with predicted biological activity [228]. Basically, in silico approaches can be categorized as structure-based modelling, in which protein structures, especially cocomplexed structures, are adopted to investigate protein-ligand interactions normally carried out by docking, and analogue-based modeling by quantitative structure-activity relationship (QSAR) and pharmacophore, in which the predictive models are derived based solely on ligand information.

Importantly, in silico methods are a logical extension of controlled in vitro experiments to shorten massive screens via high throughput methods. There are the natural results of the explosive increase in computing power available to the research scientist [229]. Several molecular models have selected protein-ligand complexes from the protein data bank (PDB) database, and the performance of docking has been evaluated by software including LigandFit, Glide, Gold, MOE Dock, AutoDock, and Surflex-Dock [14]. Recently, the polyphenol primary structure of catechin, hesperetin, and kaempferol have been found to express stable chemical characters that can have inhibitory effects on α-glucosidase and α-amylase enzymes [230]. Interestingly, polyphenol families provide an enormous resource to explore novel α-glucosidase and α-amylase inhibitors because their biological properties are abundant in functional foods represent supplementary and nutraceutical use for DM treatment [230]. The objective of this review is to investigate in silico strategies to screen polyphenol-containing herb plants that can inhibit α-glucosidase and α-amylase enzymes. In silico approaches to find novel α-glucosidase and α-amylase inhibitors from natural compounds to treat T2DM have been demonstrated by Esmail et al. (2019) [231], in that polyphenols can reduce hyperglycemia and improve acute insulin secretion or insulin sensitivity [13]. Inhibition of α-glucosidase and α-amylase can reduce the impact of dietary carbohydrate on blood glucose level [49]. Moreover, it has been observed that polyphenols play an important role in decreasing insulin resistance in vitro and improving glucose homeostasis in vivo [51]. Polyphenols such as flavonoids, phenolic acid, and stilbene have been implicated in the treatment of various human disorders [128] including diabetes [232]. As such, it is plausible to expect that polyphenols can be an important source of α-glucosidase and α-amylase inhibitors to treat T2DM. Model simulation have predicted candidates in relation to the binding of polyphenols to the three-dimensional structure of both enzymes [129]. Inhibition of carbohydrate metabolism during saccharide digestion, via α-glucosidase and α-amylase inhibition, can play a major role in T2DM treatment associated with docking studies to determine enzyme inhibition based on the free energy of binding, since hydrogen bond interactions are when binding α-glucosidase and α-amylase [233]. 

### 5.1. Docking

In general, several elements should be included in a homology template: the nature of the docked ligands; the docking program; the molecular dynamics (MD) package (refining the docked poses), and post docking calculations (Force field for MD) in the procedure of molecular docking. The procedure should involve the following: (i) three-dimensional structures of target protein are explored in the PDBor “Uniprot” website; (ii) three-dimensional structure of anticipated small molecules are retrieved from PubChem; (iii) the H_2_O of target proteins and small molecules are removed, and (iv) molecular docking is conducted by the “GOLD” platform.

A selected target such as α-glucosidase protein structure is constructed by homology modeling based on the protein structure of oligo-1,6-glucosidase from *B. cereus* (PDB 1UOK). Next, various nature of docked ligand derivatives are docked using the program CDOCKER, followed by molecular dynamics (MD) calculations by GROMACS with AMBER03 force field for refinement. The correlation coefficient between the observed Ki values and calculated interaction energies is 0.89, suggesting that binding modes are plausible [234]. Various crystal structures of enzymes, whose PDB codes are 2ZJ3 [235], 3TOP [236], 3AJ7 [237], 3A47 [238], 3A4A [239], 3AHX [239], and 3CZJ [240] have been published. Some protein structures have been adopted to conduct docking studies, as listed in Table 3, along with a selection of docking packages. PDB is the enzyme from *Homo sapiens* but specific proteins are not determined due to functional protein divergence of gene sets of these protein against α-glucosidase and α-amylase [27]. Inhibiting the activity of these two enzymes can mediate and control postprandial hyperglycemia and reduce developing diabetes [241]. The interaction between betulinic acid (BA) and α-glucosidase is obtained from the oligo-1,6-glucosidase structure (PDB ID: 3AJ7) using the CDOCKER module of Discovery Studio. The predominate factors in determining BA-α-glucosidase are hydrophobic interactions and hydrogen bonds [242]. Moreover, the oligo-1,6-glucosidase structure (PDB ID: 3AJ7) was used as the template by Ding et al. (2018) to build an α-glucosidase homology model to study the inhibitory mechanisms of oleanolic acid and ursolic acid using LeDock (available at http://www.lephar.com/software.htm, accessed on 4 July 2019). Oleanolic acid can form hydrogen bonds with Ser295 and Glu270, whereas ursolic acid can establish hydrogen bonds with Gln66 andGln67. Zeng et al. (2019) docked galangin into a homology-built α-glucosidase based on oligo-1,6-glucosidase from *S. cerevisiae* (PDB ID: 3A4A) using AutoDock (available at http://autodock.scripps.edu/accessed on 12 August 2019). It was observed that galangin can form hydrogen bonds with Leu313 and Glu411 of α-glucosidase. Interestingly, it was also found that α-glucosidase can undergo conformation change upon binding with galangin, leading to decreased enzymatic activity by hindering substrate entrance consistent with the observation made by Ding et al. (2018) (*vide supra*) [243]. In silico methods are expanded for predicting potent receptor targets, including AutoDock Vina, VMD Quantum Chemistry Visualization, Maestro 10.2 software package (Maestro is a front-end GUI), PyMol software (PyMol is a visualizer), MOE-Dock module (v.2011.10), Model Scoring of GB/VI test, force field AMBER, SCM model, SiteMap (Schrodinger Release 2018-1: SiteMap), and Pardock (Table 3).

More specifically, e.g., AutoDock was used to dock quercetin compounds into the α-amylase structure extracted from the human glutamine-complexed structure (PDB: 2ZJ3) using the binding scoring function. The binding strength between enzymes hit compounds were identified. The docking results presented novel inhibitors that were obtained according to the different criteria of docking program. Scoring functions for docking are potent approximate mathematical protocols applied to predict the strength of hydrogen interactions and binding affinity [244]. Importantly, scoring can predict robust intermolecular interactions [248]. In addition, scoring function focuses on nonbonded terms of a molecular force-field [249]. Recently, Etsassala (2019) found abietane diterpenes from *Salvia Africana-lutea* act as novel α-glucosidase and α-amylase inhibitors that exhibit strong antioxidant and anti-diabetic activities [250]. In vitro methods have characterized their chemical structure that consists of terpenoids including mangiferolic acid, cycloartenol, and ambonic acid [250,251]. Moreover, a novel in silico study revealed a new class of triazoloquinazolines that are potent α-glucosidase inhibitors [252]. Lastly, key residuals are revealed when Phloretin is docked into the protein using Surflex-Dock (Tripos Inc., St. Louis, MO, USA). It is found that phloretin can interact with Asp69, Asp215, Arg442, Gln353, and Asn350 to form five hydrogen bonds [253]. In another example, the α-amylase structure was excerpted from RCSB Protein Data Bank (http://www.rcsb.org/pdb accessed on 4 July 2019) (PDB: 1B2Y), whereas the α-glucosidase structure was built based on the protein structure of oligo-1,6-glucosidase from *S. cerevisiae* (PDB ID: 3A4A). The docking simulations were carried out by the Molecular Operating Environment (MOE, Chemical Computing Group, Montreal, Canada). It was found that the most potent FG forms hydrogen bonds with His 201, Glu 233, Asp 197, Gln 63, Trp 59 of α-amylase, and the most potent α-glucosidase inhibitor interacts with Thr 306, Asp 352, Arg 213, Glu 277, Asp 215, Arg 442 of the target protein through hydrogen bonds [253]. Quintero-Soto et al. (2021) selected the most active alcalase hydrolyzate fraction from eight chickpea (*Cicer arietinum* L.) samples to align with the complex *α*-amylase enzyme (PDB code: 1HNY) and *α*-glucosidase enzyme (PDB: 5NN8) using GRAMM-XProtein-Protein, followed by molecular dynamic simulation using the Rosetta FlexPepDock. It was found that inhibitors can bind to both enzymes by electrostatic interaction, hydrogen bonds, and hydrophobic interactions. Furthermore, sulfur-X bonds were found in the inhibitor-*α*-glucosidase interaction [254]. Swaraz et al. (2021) selected the crystal structures of α-amylase (PDB code: 1B2Y) and α-glucosidase (PDB code: 5NN8) to dock phenolic compounds from *Blumea laciniata* (Roxb.) DC. by AutoDoc Vina. Unlike the other molecular docking studies, the conformations of docked ligands were searched by the Lamarckian genetic algorithm prior to docking. It was found that Van der Waals interactions, hydrogen bonds, halogen bonds, and π–π interactions were involved in the interactions between inhibitors and enzymes. The in vitro results indicated that borassoside E, protodioscin, and diosgenin were the most potent inhibitors, whereas in silico calculations suggested otherwise [255]. Molecular docking or virtual screening has demonstrated appealing advantages, including low error level, greater stabllty and operability, wide application, low-cost and capability to scale up easily. Several limitations have been found in applications of molecular docking including poor synergistic computational models, poor quality datasets, and poor standardization, poor accurate scoring functions, model interpretation issues, issues with multi-domain proteins, and assessment of multi-drug effects. 

### 5.2. Pharmacophore Models

A pharmacophore model is derived from the most potent ligand-protein structure by Discovery Studio to give rise to chemical features including two hydrogen bond donors (HBDs) and two hydrophobic groups (2 PHs). Pharmacophore modeling that can be classified as ligand and structure-based approaches has become a major tool in drug discovery [256] and has been extensively used in virtual screening [257]. The objective of pharmacophore modeling is to find chemical features responsible for a specific biological activity among potent ligands [258]. Therefore, the use of appropriate modeling for screening drugs for T2DM is important in evaluating the interaction between the receptor and ligand, defined as essential geometric arrangement of atoms or functional groups necessary to produce a given biological response [259]. For instance, Teresa et al. (2015) used Ligandscout [260], which is a ligand-based pharmacophore modeling package to identify one of the most important structural properties that can prevent the increase of blood glucose levels. Thus in modern medicinal chemistry it is necessary to find therapeutic agents for T2DM treatment [261]. 

Gerhard et al. (2005) developed multiple pharmacophore models based on different binding modes using *LigandScout* [262], and observed different pharmacophore models comprised of different chemical features. The built models were further adopted to virtually screen a number of commercially available chemical databases, totaling ca. 1.4 million compounds. The selected hit compounds were then docked into the α-amylase structure (PDB code: 3OLE) using *Gold*. Unlike most docking studies, in which the pose selection generally relies on scoring function, the docked poses were selected based on the chemical features derived from the acarviostatin II03 complexed structure in this study. The final hit compounds showed α-amylase inhibitory activities with assayed IC_50_ values of tens of micromoles. This study clearly illustrated the synergy between structure and analogue-based modeling, as well as in vivo assays and in silico approaches.

Pharmacophore modeling can be used in conjunction with molecular docking and molecular dynamics (MD) in some cases [263]. They are suitable for identifying the treatment of T2DM as a known anti-pharmacophore was generated to remove all potential agonists from the screening database [264]. For example, PPAR-α/γ agonists can regulate glucose metabolism including hyperglycemia and insulin resistance [265]. Thus, alternative therapeutics for antidiabetic drugs can use this modeling to arrange chemical features and some elements of drug design such as the absence of structural data for the target enzyme-linked receptor [266]. Lee et al. (2014) discovered sulfonamide chalcone derivatives from *Saccharomyces cerevisiae* as a novel class of non-saccharide compounds that can potentially inhibit α-glucosidase by molecular docking and MD simulation [267]. Interestingly, oleanonic acid and other components of *P. lentiscus* oleoresin are new partial PPARγ agonists unveiled by a pharmacophore hypothesis to treat T2DM [268]. Moreover, pharmacophore models were adopted to identify stilbene derivatives as a class of α-glucosidase competitive inhibitors [266] and sulphonamide chalcone derivatives as a new class of compounds to treat T2DM [269]. Pharmacophore also have long been studied with α-amylase to control diabetes and found to have good binding affinity [269]. Pharmacophore packages and computer software include Discovery Studio, LigandScout, Phase, MOE–Pharmacophore Discovery, ICM-Chemist, ZINCPharmer, and Pharmit model; pharmacophores are used to determine potent features of one or more molecules with the same biological activity [270]. Qualitative or quantitative studies can predict qualitative and quantitative properties and can be used for identification through virtual screening or in silico models [271]. Findings related to the docking studies, and molecular docking are used in computer-aided drug design approaches related to structure-based 3-D pharmacophore because they can predict free energy and scoring schemes to test PDB binding [271]. 

The major advantages of pharmacophore models are virtual screening of a large database, no need to know the binding site of the ligands for the target protein, the design and optimization of drugs, scaffold-hopping, 2-D structural representation, all with a comprehensive and editable approach. However, some limitations are that 2D pharmacophore is less accurate than 3D pharmacophore, no interactions with the proteins, and sensitivity to physicochemical features.

### 5.3. QSAR Model

The quantitative structure-activity relationship model (QSAR model) is a classification model used in the chemical and biological sciences and engineering. QSAR shows biological activity which can be expressed quantitatively and can be used to predict the model response of other chemical structures [272]. The QSAR model has function that identifies chemical structures for drug discovery that could have inhibitory effects on specific targets with low toxicity (nonspecific activity). Of special interest is the prediction of partition coefficient log *P*, which is an important measure used in identifying drug likeness according to Lipinski’s Role of Five [273]. QSAR schemes, which are mathematically designated to map chemical characteristics with biological activity, have been extensively adopted to predict α-glucosidase and α-amylase inhibitors [274]. QSAR studies include ligands with their binding sites, inhibition constants, rate constants and other biological end points, in addition molecular to properties such as lipophilicity, polarizability, electronic, and steric properties or with certain structural features [275]. The model attempts to find consistent relationships between the variations in the values of molecular properties and the biological activity of a series of compounds which can then be used to evaluate the properties of new chemical entities [276]. 

One study used the QSAR model to search natural α-amylase and α-glucosidase inhibitors of all collected compounds, including active α-amylase and α-glucosidase inhibitors from ChEMBl, and inactive α-amylase and α-glucosidase inhibitors from DrugBank; 640 and 214 compounds were divided into the training set and validation set for the α-amylase inhibition model development, respectively, and 1540 and 515 compounds into the training set and validation set for the α-glucosidase inhibition model development. Descriptor enumerations were carried out by Dragon, descriptor selection was done by linear discriminant analysis (LDA), and the classification models were built by the classification tree (CT) algorithm. The best derived model showed a very high level of predictivity (>95% accuracy in the training set, 86.80% in the test set, and 85.32% by the10-fold cross validation) [277].As we know, the advantages of predicting biological activity with QSAR modelling includes a large number of compounds with little to no prior experimental data on activity, molecular properties that may be worth investigating further, chemical waste is not generated when performing in silico predictions, the procedure reduces the need for testing on animals and/or on cell cultures, and saves time. Disadvantages of predicting biological activity with QSAR modelling include no in-depth insight on the mechanism of biological action, and some risk of highly inaccurate predictions of pharmacological or biological activity.

## 6. Comparing In Vitro (Enzymatic, Cellular) and In Vivo Advantages and Disadvantages of In Silico Modeling Applications in T2DM)

A variety of in vitro and in vivo assays have been proposed to find novel therapeutic agents targeting various putative molecular targets (*vide supra*) for T2DM treatment [278]. Drug discovery processes are important and include variety of activities using assay models (in vitro, in vivo, and in silico) [279]. In vitro and in vivo tests for T2DM can also evaluate the toxicity of drugs or compounds in development and the modified activity, and bioavailability of herbal medicine compounds [280]. Importantly, preliminary study includes protein-ligand interactions comparable to the lock and key principle [281]. The major potent force for binding is hydrophobic interaction, while in silico modeling can be helpful to identify drug target via bioinformatics tools [282]. The advantage of in silico methods is to explore the target structures as possible active sites to generate candidate molecules with results related to their binding affinity and hydrogen interaction [283]. The disadvantage of in silico methods is that the binding mode and score function has been extensively tested with multiple ligands for binding mode prediction, and affinity prediction and many scoring functions might yield inaccurate predictions with less precision compared to in vitro and in vivo methods [284]. Hence, enzymatic or cellular assays, animal models, and in silico modelling are essential for developing new anti-diabetic agents and alternative therapeutics in the future. Moreover, use of techniques and algorithms “in silico” is a good way to identify new molecules as subjects for in vitro cell studies, animal in vivo tests for validation and finally in human clinical trials for filing drug licenses.

## 7. Perspectives of In Silico Modelling for Discovery and Development of Anti-Diabetes Drugs

Drug repositioning (DR) is the process of classifying new indications for approved drugs and can substantially expedite drug discovery and development based on the fact that their toxicity issues have been evaluated previously [285]. DR can reduce time and cost because it takes advantage of drugs already in clinical use for other indications, or drugs that have passed phase I safety trials but failed to show efficacy for the intended diseases. In silico drug discovery methods, and the development of antidiabetic drug repurposing has become an important factor in new drug discovery. Several computational approaches that help us to uncover new antidiabetic drug opportunities and discovery process have been screened or adapted from previous applications [286]. Accordingly, identification of new drugs is expected to help predict new drug-targets, [228]. In silico approaches are capable of complement and integrating with each other in drug repurposing and will result in drugs for the future [287]. Computational (in silico) methods have been developed and widely applied to pharmacological hypothesis development and tests, including database, structure-activity relationships (SAR), pharmacophores, molecular docking, and super-speed computer tools [288]. Drug design and development for T2DM are still in early stages of management. The conventional target and structure-based methods can be linked toward therapeutic mechanism of T2DM treatment [257]. In contrast, several approaches in silico have the advantage of fast speed and low cost, and has been receiving more attention worldwide; the disadvantage being over-estimation of binding affinity and arbitrarily choosing non-bonded cut off terms [245]. Currently, we have performed in silico assays to accelerate new discoveries from herbal or natural compounds and confirmed the validation of preclinical levels. These include: α-amylase and α-glucosidase activities suppressed by Garcinia linii extracts, including syringaldehyde, via docking, and further confirmed by in vitro (cell) and in vivo (diabetic mice) studies [289]; by the mixture of extracts (purple onion, cinnamon, and tea) via docking, and further confirmed by in vitro (enzyme) and in vivo (diabetic mice) studies [290]; by γ-mangostin via docking and further confirmed by in vitro (cell, enzyme) and in vivo (diabetic mice) studies [291]; by syringaldehyde via docking and further confirmed by in vitro (enzyme, organ culture) and in vivo (diabetic mice) [292] studies; and by curcumin, antroquinonol, HCD, docosanol, tetracosanol, rutin, and actinodaphnine via virtual screen and further confirmed by in vitro (cell) and in vivo (diabetic mice) studies [293]. Remarkably, drug design is a process in which new leads (efficacy drugs) are discovered which have therapeutic benefits in antidiabetic drugs and can have potential effects on the management of T2DM in human clinical trials [246,247].

## Figures and Tables

**Table 1:** Molecular targets of antihyperglycemia therapy drug.

Class	Mechanism of Action	Generic Name	Side Effects
**α-Glucosidase and α-amylase inhibitors**	Retards carbohydrate digestion, extends overall digestion time and diminishes glucose level absorption [26].	Acarbose, Miglitol [27]	Mild stomach pain, gas or bloating, constipation, diarrhea [28].
**Sodium glucose linked transporter-2 (SGLT-2) inhibitors**	Inhibits SGLT2 in proximal convoluted tubule (PCT) to block reabsorption of glucose and facilitate its secretion in urine [29].	Dapagliflozin, Canagliflozin, Sitagliptin [30].	Upset stomach, diarrhea, headache [31].
**Dipeptidyl peptidase 4 (DPP-4) inhibitors**	Blocks DPP-4 activity in peripheral plasma, that inhibits the incretin hormone glucagon-like peptide (GLP)-1 in the peripheral circulation [32].	Sulfonylureas, Thiazolidinediones, Biguanides [33]	Hunger, weight gain, skin reaction [34]
**Peroxisome proliferator activated receptor-γ (PPARγ)**	Diminishes triglyceride level related to regulation of energy homeostasis [35].	PPAR γ agonist, RXR (Retinoid X receptors) agonists (rexinids) [35].	Weight gain, fluid retention, increased risk of heart failure [36].
**Insulin receptor kinase (IRK)**	Insulin receptor as a tetrameric glycoprotein and binds to specific cell surface receptors in its target cells resulting in insulin effects on phosphorylation [37].	IRS (1, 2, 3, 4), SHC (*Src* homology 2 domain containing) [38].	Unclear whether safe or effective treatment [39].
**Insulin receptor substrate (IRS)**	Protein cytoplasmic adaptor that functions as a crucial signalling intermediates downstream of the activated cell surface [40].	IGF-1 (insulin-like growth factor 1), IGF-2, Insulin [41].	Hypotension, fluid retention, orthostatic [42].
**Glucose transporter 4 (GLUT4)**	Expressed in muscle and regulates insulin-stimulated glucose uptake within muscle tissue [43].	MET2 (Myocyte enhancer factor-2), MyoD myogenic protein [43].	Remained largely unknown [44].
**G protein-coupled receptors (GPCR)**	Works with β-cells to inhibit insulin secretion and the number of β-cell GPCRs related to insulin controlling secretion [45].	Insulin secretagogues, GLP-1 (glucagon-like peptide-1), GIP (glucose-dependent insulinotropic peptide) [45].	Vomit, diarrhea, gastrointestinal problems [46].

**Table 2:** List of polyphenol plant families that inhibit α-glucosidase and α-amylase.

Family	Enzymatic Type	Scientific Name
	α-Glucosidase inhibitor	
Theaceae		*Camellia sinensis* Ktze [128]
Myrtaceae		*Cleistocalyx operculatus* Roxb [129]
Fabaceae		*Sophora japonica* L. [130]
		*Senna surattensis* [11]
		*Alhagi camelorum* [51]
		*Neptunia oleracea* [131]
		*Peltophorum pterocarpum* [132]
Asteraceae		*Artemisia vulgaris* L. [133]
Lecythidaceae		*Careya arborea* Roxb [134]
Apiaceae		*Centella asiatica* (L.) Urb [135]
		*Eryngium foetidum* L. [136]
		*Levisticum officnale* [137]
		*Ligusticum porter* [138]
Moraceae		*Ficus racemosa* L. [16]
		*Artocarpus champeden* [139]
		*Morus alba* [140]
Myristicaceae		*Horsfieldia amygdalina* Warb [130]
Saururaceae		*Houttuynia cordata* Thunb [141]
Rubiaceae		*Paederia lanuginosa* Warb [130]
		*Cinchona succirubra* [142]
		*Hintonia latiflora; H. standleyana* [143]
Verbenaceae		*Premma corymbosa* (Burm) [144]
Euphorbiaceae		*Euphorbia thymifolia* [124]
Lamiaceae		*Perilla frutescens* (L.) Britton [145]
		*Rosmarinus officinalis* [146]
		*Zataria multiflora* [147,148]
		*Zhumeria majdae* [51]
Polygonaceae		*Polygonum odoratum* Lour [149]
Clusiaceae		*Garcinia daedalanthera* [87,150]
Scrophulariaceae		*Verbascum kermanensis* [51]
Rosaceae		*Rosa damascene* [151]
		*Sanguisorba minor* [51]
		*Sarcopotarium spinosum* L. [152]
Anacardiaceae		*Pistacia vera* [51]
Ericaceae		*Vaccinum arctostaphylus* [153]
Salvadoracae		*Salvadora persica* [154]
Zingiberaceae		*Alpinia officinarum* [155,156]
Phyllantaceae		*Antidesma bunius* Spreng [157]
Oxalidaceae		*Averrhoa bilimbi* L. [158]
		*Biophytum sensitivum* L. DC [159]
Rhizophoraceae		*Ceriops tagal* Perr. Rob [160]
		*Rhizophora mucronata* Lam [161]
Cyperaceae		*Kyllinga monocephala* Rottb [162]
Asteraceae		*Brickellia cavanillesii* [163]
		*Blumea lanceolaria* Roxb [164]
Celastraceae		*Salacia oblonga* [165]
Lamiaceae		*Scutellaria baicalensis* [166]
Cucurbitaceae		*Cucurbita pepo* L. [167]
Convolvulaceae		*Ipomoea aquatica* Forssk [168]
		*Ipomoea batatas* (L.) Lam [169]
Piperaceae		*Piper lolot* DC [130]
Brassicaceae		*Nasturtium officinale* R. Br [170]
Myrtaceae		*Eucalyptus grandis* [171]
		*E. urophylla* [171]
		*Syzygium aqueum* [172]
		*S. cumini* [173]
Meliaceae		*Azadirachta indica* [139]
Clusiaceae		*Garcinia mangostana* [174]
Sapindaceae		*Nephelium lappaceum* [175]
Vitaceae		*Vitis vinifera* [176]
Santalaceae		*Osyris alba* L. [177]
Hypericaceae		*Hypericum triquetrifolium* Turra [178]
Ericaceae		*Arbutus andrachne* L. [179]
		*Vaccinium oxycoccos* [180]
Bignoniaceae		*Oroxylum indicum* [123]
Campanulaceae		*Codonopsis pilosula* [181,182]
Geraniaceae		*Geranium collinum* [183]
Dryopteridaceae		*Dryopteris cycadina* [75,184]
Acanthaceae		*Clinacanthus nutans* [142]
Rutaceae		*Orixa japonica* Thunb [156]
	α-Amylase inhibitor	
Anacardiaceae		*Spondias pinnata* (Koenig) [185]
Myrtaceae		*Syzygium cumini* L. [186]
Zygophyllaceae		*Balanites aegyptiaca* L. [187]
Amaranthaceae		*Amaranthus caudatus* L. [188]
Theaceae		*Camellia sinensis* L. Del [128]
Fabaceae		*Galega officinalis* L. [189]
		*Tamarindus indica* L. [190]
		*Cassia auriculata* [191]
Apocynaceae		*Holarrhena floribunda* [192]
		*Melissa officinalis* L. [193]
Rubiaceae		*Mitragyna innermis* (Wild.) [189]
Lamiaceae		*Rosmarinus officinalis* L. [193]
Polygalaceae		*Securidaca longepedunculata* [194]
Asparagaceae		*Polygonatum adoratum* [195]
	α-Glucosidase and α-amylase inhibitor	
Nelumbonaceae		*Nelumbo nuciffera* Gaertn [47]
Asteraceae		*Artemisia vulgaris* L. [133]
		*Enydra fluctuans* Lour [185]
Araliaceae		*Polyscias fruticosa* (L.) Harms [196]
Myrtaceae		*Syzygium zeylanicum* (L.) DC [186]
Phyllanthaceae		*Phyllanthus amarus* [126]
		*Phyllanthus urinaria* [127]
Lamiaceae		*Ocimum basilicum* L. [125]
		*Thymus serpyllum* [197]
Meliaceae		*Khaya senegalensis* [198]
Moraceae		*Artocarpus altilis* [1,199]
Ranunculaceae		*Aconitum heterophyllum* [199]
Acoraceae		*Acorus calamus* [200]
Berberidaceae		*Berberis aristata* [199]
Cyperaceae		*Cyprus rotundus* [201]
Calophyllaceae		*Mesua ferrea* [186]
Plumbaginaceae		*Plumbago zeylanicum* [202]
Combretaceae		*Terminalia arjuna* [203]
Myrtaceae		*Brazilian cerrado* [204]
		*Eugenia dysenterica* [205]
		*Stryphnodendron adstringens* [206]
		*Pouteria caimito* [206]
		*Pouteria torta* [206]
		*Pouter ramiflora* [207]
		*Psidium guajava* L. [2]

**Table 3:** Natural compounds against α-glucosidase and α-amylase enzymes discovered via in silico approaches, listing the docking package and scoring function used in the studies.

In Silico Modeling				
Natural Compound	Plant Family	Binding Energy (Kcal/mol)	PDB ID	Hydrophobic & Hydrogen-Bond Interaction
Quercetin	*Euphorbiaceae*	−7.6	2ZJ3; *Homo sapiens*, AutoDock Vina [211], VMD Quantum Chemistry Visualization [228,244]	Ser420, Lys675, Gln421, Thr375, Ser422
Quercitrin		−9.0		
Quercetin-3-O-galactoside		−9.1		
Cosmosiin		−9.9		
Kaempferol		−7.6		
2-(4 methyl-3-cyclohexene-1-yl)-2-propanol		−5.4		Val677, Ala674, Thr375
Β-amyrine		−9.0		
Β-Sitosterol		−7.8		
Campesterol		−8.2		
Caryophyllene		−7.1		
Limonene		−4.8		
Phytol		−5.2		
Piperitenone		−5.4		
Safranal		−5.5		
Stigmasterol		−8.5		
Taraxerol		−8.9		
Euphorbol		−8.3		
24 methylene cycloartenol		−7.9		
1-O-Galloyl-beta-D-glucose		−8.0		
Corilagin		−8.9		Ser420, Lys675, Gln421, Thr375, Ser422
Baicalein	*Bignoniaceae*	−6.98	3TOP; *Homo sapiens*, *Schrodinger* Maestro [116]	Pro1327, Glu1284, Pro1405, Leu1401
Catechin		−7.70		His1584, Asp1279, Asp1526, Arg1510, Asp1157
Luteolin		−7.52		
Quercetin		−7.19		
Quinoline	*Rubiaceae*	−8.6	3AJ7; *Saccharomyces cerevisiae*, MOE-docking 2010.11software [140]	Phe177, Asp214, His279, Phe157
Benzothiazole	*Ericaceae*	−8.08	No mentioned for PDB code, 3D structure: α-glucosidase of *Saccharomyces cerevisiae*, AutodockTools 1.5.6 package [161], PyMol 1.7.6 software (http://www.pymol.org/, accessed on 19 February 2020)	Phe157, Phe310, Phe311
β-Sitosterol	*Dryopteridaceae*	−16.097	The three-dimensional structure for α-glucosidase of *Saccharomyces cerevisiae* has not yet been solved, MOE-Dock (MOE 2010.11) software [165]	Asp215, Asp352, Arg442, Gln182
β-Sitosterol3-O-β-D-glucopyranoside		−7.756		Asn415
2, 3, 5, 7-trihydroxy-2-(p-tolyl) chorman-4-one		−22.480		Arg315, Asp307, His280, Lys156, Ser240, Thr310, Tyr158
Quercetin-3-0-β-D-glucopyranoside (3/→0-3///)-β-D-Quercetin-3-0-β-D-galactopyranoside		−12.931		Arg442, Tyr158
5, 7, 4/-Trihydroxyflavon-3-glucopyranoid		−15.752		Asp242, Lys156, Pro312, Tyr158
2,6-diethylpiiperidine-3,4,5-triol	*Campanulaceae*	−6.1790	3A47; *Saccharomyces cerevisiae*, MOE-Dock module (v.2011.10), Model Scoring of GB/VI test, The force field AMBER99 [143]	Lys155, Glu304, Arg312, Asn153
2-ethyl-6-methylpiperidine-3,4,5-triol		−8.8493		
6-ethyl-2-(hydroxymethyl)piperidine-3,4-diol		−6.9539		
1,2,4-tri-O-gal-loyl-β-D-glucopyranose	*Geraniaceae*	−8.7	3AHX; *Clostridium cellulovorans*, The SCM model, SiteMap (Schrodinger Release 2018-1: SiteMap, Schrodinger, LLC, New York, NY, 2018) [163]	Asp232, Ser235, Asn314, Glu426
Kaempferol-3-O-α-rhamnopyranoside		−9.4		Asp214, Asn241, Val277
Kaempferol-3-O-α-arabinofuranoside		−9.2		Asp68, Asp214, Thr215, Glu276, Asp408
Quercetin-3-O-β-glucuronopyranoside		−9.8		Asp68, Asp214, Arg312, Asp349, Gln350
Quercetin-3-O-α-arabinofuranoside		−5.4		Asp232, Asp429
Kuwanon L	*Moraceae*	−8.4412	3A4A; *Saccharomyces cerevisiae*, Agilent Masshunter software Ver. B.04.00, The Molecular Operating Environment (MOE.2009.10) software [131]	Asp 69, Asp215, Asp352, Asp307
Mulberrofuran G		−8.4634		
Sanggenon C		−8.4291		Asp69, Asp352, Asp215, Glu277
Moracenin D		−8.3188		Asp 69, Asp215, Asp352, Asp307
Mortatarin C		−5.4358		No interaction
Sanggenon G		−9.2855		Asp69, Asp352, Asp215, Glu277, Phe178
Sanggenon O		−8.9427		Asp69, Asp352, Asp215, Glu277
Sanggenol A		−7.7639		No interaction
Sanggenon W		−8.4194		Asp 69, Asp215, Asp352, Asp307
5′-Geranyl-5,7,2′,4′-tetraphydroxy flavone		−8.2431		No interaction
Nigrasin F		−8.0232		Asp 69, Asp215, Asp352, Asp307
Sanggenol G		−8.7875		Asp69, Asp352, Asp215, Glu277
Mortatarin B		−5.9508		Asp 69, Asp215, Asp352, Asp307
4,6,8-Megastigmatrien-3-one	*Acanthaceae*	−7.47	3A4A; *Saccharomyces cerevisiae*, 2PQR; *Saccharomyces cerevisiae*, AutoDock Tools, Biovia Discovery Studio (San Diego, CA, USA, USA), PyMOL^TM^ 1.7.4.5 (Schrodinger, LLC, New York, NY, USA) [245]	Asn259, Hid295
N-Isobutyl-2-nonen-6,8-diynamide		−5.54		Lys156
1′,2′-bis(acetyloxy)-3′,4′-didehydro-2′-hydro-β,ψ-carotene		−10.19		Arg335
22-acetate-3-hydroxy21(6-methyl-2,4-octadienoate)-olean-12-en-28-oic acid.		−8.31		Gly209
Polyhydroxy pyrrolidines	*Rutaceeae*	−2.4	3CZJ; *Escherichia coli*, Pardock (http://scfbio-iitd.res.in/dock/paradock.jsp, accessed on 19 February 2020), Accelrys and AutoDock software (AutoDock v.4.2.6, San Diego, CA, USA) [246,247]	No interaction
Tosyl		−3.1		Asp229, Asp231

## Data Availability

Not applicable.
